# Efficient bioconversion of 2,3-butanediol into acetoin using *Gluconobacter oxydans* DSM 2003

**DOI:** 10.1186/1754-6834-6-155

**Published:** 2013-10-31

**Authors:** Xiuqing Wang, Min Lv, Lijie Zhang, Kun Li, Chao Gao, Cuiqing Ma, Ping Xu

**Affiliations:** 1 State Key Laboratory of Microbial Technology, Shandong University, Jinan 250100, People’s Republic of China; 2 State Key Laboratory of Microbial Metabolism, School of Life Sciences and Biotechnology, Shanghai Jiao Tong University, Shanghai 200240, People’s Republic of China

**Keywords:** 2,3-butanediol, Acetoin, *Gluconobacter oxydans*, Bioconversion

## Abstract

**Background:**

2,3-Butanediol is a platform and fuel biochemical that can be efficiently produced from biomass. However, a value-added process for this chemical has not yet been developed. To expand the utilization of 2,3-butanediol produced from biomass, an improved derivative process of 2,3-butanediol is desirable.

**Results:**

In this study, a *Gluconobacter oxydans* strain DSM 2003 was found to have the ability to transform 2,3-butanediol into acetoin, a high value feedstock that can be widely used in dairy and cosmetic products, and chemical synthesis. All three stereoisomers, *meso*-2,3-butanediol, (2*R*,3*R*)-2,3-butanediol, and (2*S*,3*S*)-2,3-butanediol, could be transformed into acetoin by the strain. After optimization of the bioconversion conditions, the optimum growth temperature for acetoin production by strain DSM 2003 was found to be 30°C and the medium pH was 6.0. With an initial 2,3-butanediol concentration of 40 g/L, acetoin at a high concentration of 89.2 g/L was obtained from 2,3-butanediol by fed-batch bioconversion with a high productivity (1.24 g/L · h) and high yield (0.912 mol/mol).

**Conclusions:**

*G. oxydans* DSM 2003 is the first strain that can be used in the direct production of acetoin from 2,3-butanediol. The product concentration and yield of the novel process are both new records for acetoin production. The results demonstrate that the method developed in this study could provide a promising process for efficient acetoin production and industrially produced 2,3-butanediol utilization.

## Background

2,3-Butanediol is a platform and fuel biochemical (<US$1/kg) that can be produced by biotechnological routes. With a high heating value of 27,200 J/g, it can be used as a liquid fuel or fuel additive [1-3]. Many microorganisms including *Bacillus*, *Klebsiella*, *Enterobacter*, *Saccharomyces*, and *Serratia* have been used to efficiently produce 2,3-butanediol [4-10]. Although some efficient and economical 2,3-butanediol fermentation processes have been established in laboratory studies [11-15], it has not been produced in a large scale. The reason is because a sizable derivative process for this chemical has not yet been developed until now. Hence, the development of improved derivative processes of 2,3-butanediol would be a prerequisite for commercial utilization of industrially produced 2,3-butanediol.

**Figure 1:**
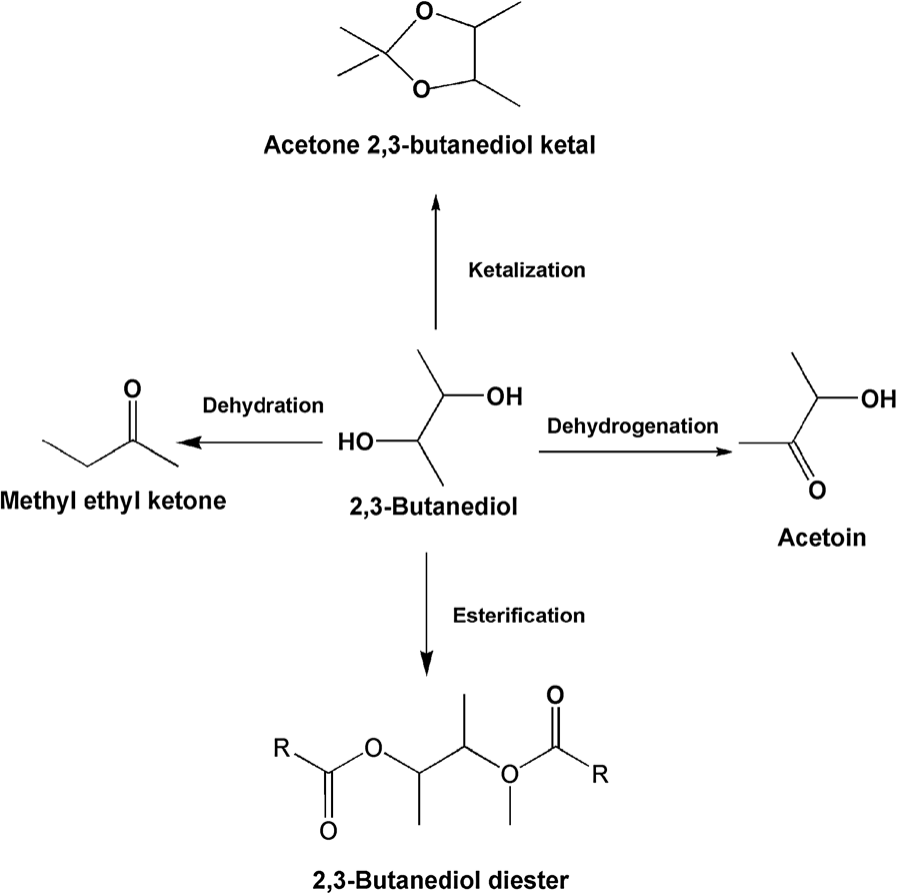
Derivatives of biologically produced 2,3-butanediol through different chemical reactions.

Several interesting chemical reactions, such as dehydration, dehydrogenation, ketalization, and esterification, could be used for the preparation of 2,3-butanediol derivatives (Figure 1). Among those derivative processes of 2,3-butanediol, only dehydrogenation of 2,3-butanediol to produce acetoin could be performed by biotechnological routes. Acetoin is a high value product that can be widely used not only in dairy products, but also in cosmetics, pharmaceutical, and chemical synthesis [16-19]. It is one of the 30 platform chemicals that are given priority to their development and utilization by the US Department of Energy. Thus, numerous studies have been executed to find an effective biocatalytic process to produce acetoin from 2,3-butanediol. For example, Yamada-Onodera *et al*. reported that 8.4 g/L of acetoin was obtained from 2,3-butanediol after 24 hours of incubation with recombinant *Escherichia coli* expressing glycerol dehydrogenase [20]. A recombinant *E. coli* strain that coexpressed (2*R*,3*R*)-2,3-butanediol dehydrogenase and NADH oxidase was also constructed, and the highest yield of acetoin was found to be 36.7 g/L [21]. However, in these biocatalytic processes, biocatalysts must be cultivated, separated, and washed before being used in the production of acetoin; such a complicated operation presents a significant drawback for the application of the method.

Production of acetoin using 2,3-butanediol as the sole carbon source does not require the separation of biocatalysts from growth medium. It is an interesting concept, but unfortunately acetoin can be metabolized by numerous microorganisms [22-25]. A microorganism that could directly produce acetoin from 2,3-butanediol through bioconversion has never been reported. Thus, it would be desirable to find an effective microorganism for the direct production of acetoin from 2,3-butanediol.

In this study, *Gluconobacter oxydans* DSM 2003, an obligate aerobic Gram-negative bacterium, was confirmed to have the ability to produce acetoin from 2,3-butanediol. After optimization of reaction conditions, production of acetoin from 2,3-butanediol using *G. oxydans* DSM 2003 was acquired. The process presented in this study could provide a promising alternative for the value-added utilization of biotechnologically produced 2,3-butanediol from biomass.

## Results and discussion

### *G. oxydans* DSM 2003 has the capacity for acetoin production from 2,3-butanediol

*G. oxydans* has a respiratory metabolism characterized by incomplete oxidation of sugars, alcohols, and acids. The partially oxidized products (aldehyde, ketone, and organic acid) are rapidly excreted into the medium. This property makes *G. oxydans* an important biocatalyst for industrial use [26-28]. In a previous study, many substrates including glycerol, meso-erythritol, 1,3-butanediol, and 2,3-butanediol could be oxidized by the membrane-bound polyol dehydrogenase (GOX 0854 and GOX 0855) in *G. oxydans* 621H [29]. Homologues of GOX 0854 and GOX 0855 were present in other *G. oxydans* strains, such as *G. oxydans* H24, *G. oxydans* DSM 7145, and *G. oxydans* IFO 3255 [30-32]. Thus, most strains of *G. oxydans*, such as *G. oxydans* DSM 2003, might have the metabolic potential to directly produce acetoin from 2,3-butanediol.

**Figure 2:**
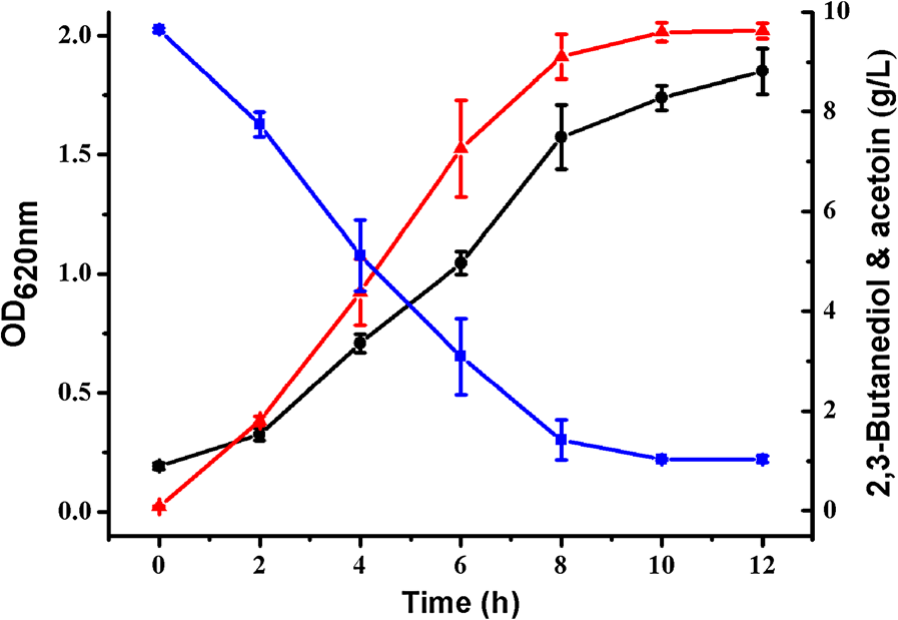
**Time course of *G. oxydans *DSM 2003 growth in media containing 10 g/L 2,3-butanediol.** The experiments were conducted in 300 mL shake flasks containing 50 mL of medium at 30°C. Biomass (black circle); 2,3-butanediol (blue square); and acetoin (red triangle).

To determine whether the *G. oxydans* DSM 2003 has the capability to produce acetoin from 2,3-butanediol, the strain was cultured in a medium containing 20 g yeast extract, 1.5 g (NH_4_)_2_SO_4_, 1.5 g KH_2_PO_4_, and 0.5 g MgSO_4_ 7H_2_O in 1 L of distilled water. This medium was supplemented with 10 g/L 2,3-butanediol. The flask experiment was conducted in 300 mL shake flasks containing 50 mL fresh medium for 12 hours at 200 rpm and 30°C. As shown in Figure 2, 9.6 g/L acetoin was obtained in 12 hours. However, little growth of *G. oxydans* DSM 2003 and no production of acetoin were detected in the medium that contained 20 g/L yeast extract (Additional file 1: Figure S1A). *G. oxydans* DSM 2003 was also cultured in the medium containing 10 g/L glycerol, a good carbon source for the growth of the strain. Although a higher growth of the strain was detected, no production of acetoin was detected in the medium containing 10 g/L glycerol (Additional file 1: Figure S1B). On the other hand, homologues of key enzymes in 2,3-butanediol synthesis pathway (α-acetolactate synthase and α-acetolactate decarboxylase) were absent in the genome sequenced *G. oxydans* strains including *G. oxydans* 621H, *G. oxydans* H24, and *G. oxydans* WSH-003 [32-34]. Thus, there might not be a 2,3-butanediol producing pathway in *G. oxydans* DSM 2003. Acetoin was produced by a direct dehydrogenation reaction of 2,3-butanediol in the medium.

### All three stereoisomers of 2,3-butanediol are utilized by *G. oxydans* DSM 2003

2,3-Butanediol has three stereoisomers including *meso*-2,3-butanediol, (*2R*,*3R*)-2,3-butanediol, and (*2S*,*3S*)-2,3-butanediol. Different microorganisms produce different stereoisomers of 2,3-butanediol. For example, strains of *Bacillus*, such as *Bacillus licheniformis*, and *Paenibacillus polymyxa* produce (2*R*,3*R*)-2,3-butanediol as the major product [7,35]. *Serratia marcescens* produces *meso*-2,3-butanediol as the major product [11]. Other strains including *Klebsiella pneumoniae*, *Klebsiella oxytoca*, and *Enterobacter cloacae* produce *meso*-2,3-butanediol and (2*S*,3*S*)-2,3-butanediol as the major products [4,5,9]. In this study, a commercial 2,3-butanediol, which contained 15.9% (2*R*,3*R*)-2,3-butanediol, 76.1% *meso*-2,3-butanediol, and 8.0% (2*S*,3*S*)-2,3-butanediol, was used as the carbon source for *G. oxydans* DSM 2003. After the bioconversion process, the stereoisomeric composition of 2,3-butanediol was analyzed by gas chromatography (GC) with a flame ionization detector and a fused silica capillary column.

All three stereoisomers of 2,3-butanediol including *meso*-2,3-butanediol, (2*R*,3*R*)-2,3-butanediol, and (2*S*,3*S*)- 2,3-butanediol could be utilized by *G. oxydans* DSM 2003. Both (3*S*)-acetoin and (3*R*)-acetoin were the final products of the bioconversion process. Thus, the 2,3-butanediol produced by the reported microorganisms could be used by *G. oxydans* DSM 2003 for acetoin production.

### *G. oxydans* DSM 2003 catalyzes 2,3-butanediol into acetoin with stereoselectivity

Among all of the 2,3-butanediol producing strains, 2,3-butanediol was produced by NAD-dependent 2,3-butanediol dehydrogenase, catalyzing the stereoselective reduction of acetoin [2]. Several 2,3-butanediol dehydrogenases with different stereospecificities have been previously studied. 2,3-Butanediol dehydrogenase could also catalyze the oxidation of 2,3-butanediol to produce acetoin. For example, (2*R*,3*R*)-2,3-butanediol dehydrogenase in *Bacillus subtilis*, *Saccharomyces cerevisiae*, and *Paenibacillus polymyxa* can catalyze the stereospecific oxidation of (2*R*,3*R*)-2,3-butanediol and *meso*-2,3-butanediol to (3*R*)-acetoin and (3*S*)-acetoin, respectively [36-38]. *meso*-2,3-Butanediol dehydrogenase in *S. marcescens* H30 can catalyze the stereospecific oxidation of (2*S*,3*S*)-2,3-butanediol and *meso*-2,3-butanediol to (3*S*)-acetoin and (3*R*)-acetoin, respectively [39]. To identify the stereoselectivity of 2,3-butanediol dehydrogenase in *G. oxydans* DSM 2003 that catalyzes the oxidation of 2,3-butanediol, biotransformation with 2,3-butanediol as the substrate and whole cells of *G. oxydans* DSM 2003 as the catalyst was conducted. After accomplishing the reaction with *meso*-2,3-butanediol, (2*R*,3*R*)-2,3-butanediol, or (2*S*,3*S*)-2,3-butanediol as the substrate, the mixture was centrifuged and the concentrations of (3*S*)-acetoin and (3*R*)-acetoin in the supernatant were analyzed by GC, respectively.

When (2*R*,3*R*)-2,3-butanediol was used as the substrate, (3*R*)-acetoin was the major product detected. Accordingly, (3*S*)-acetoin was, as expected, the major product obtained from (2*S*,3*S*)-2,3-butanediol. Furthermore, (3*S*)-acetoin could be obtained from *meso*-2,3-butanediol. On the other hand, as shown in Additional file 2: Figure S2A and Figure S2B (analyzed by HPLC), (3*S*)-acetoin and (3*R*)-acetoin could not be further transformed into diacetyl by *G. oxydans* DSM 2003, which is similar to the situation in most of the 2,3-butanediol producing strains.

Chiral acetoin is widely used to synthesize novel optically active α-hydroxy ketone derivatives and liquid crystal composites. Numerous biocatalytic processes for the production of chiral acetoin have been reported [40,41]. (2*R*,3*R*)-2,3-Butanediol and *meso*-2,3-butanediol could be easily produced by *P. polymyxa* and *S. marcescens*, respectively [7,11]. Due to the high stereoselectivity in the *G. oxydans* DSM 2003 catalyzed 2,3-butanediol oxidation, this strain might also provide a promising alternative for the production of (3*S*)-acetoin and (3*R*)-acetoin.

### *G. oxydans* DSM 2003 constitutively expresses enzymes in 2,3-butanediol oxidation

In *G. oxydans* 621H, the polyol dehydrogenase (GOX 0854 and GOX 0855) exhibited 2,3-butanediol dehydrogenase activity [29]. This enzyme was reported as a membrane-bound protein and uses ubiquinone as the native electron acceptor. To identify whether *G. oxydans* DSM 2003 has a similar 2,3-butanediol dehydrogenase activity, a whole-cell 2,6-dichlorophenolindophenol (DCPIP) assay was used [29]. Corresponding to the result of biotransformation experiments, *meso*-2,3-butanediol, (2*R*,3*R*)-2,3-butanediol, and (2*S*,3*S*)-2,3-butanediol could be oxidized by whole cells of *G. oxydans* DSM 2003, implying the presence of a 2,3-butanediol dehydrogenase activity in the strain.

To assess the expression of 2,3-butanediol dehydrogenase activity, *G. oxydans* DSM 2003 was cultured with different carbon sources, and the specific activities of 2,3-butanediol dehydrogenases were examined. The specific activities of the enzymes in cells grown on 2,3-butanediol were similar to those of cells grown on glucose, glycerol, and sorbitol (Additional file 3: Table S1). This result is consistent with polyol dehydrogenase in *G. oxydans* 621H, whose expression was also constitutive [29]. However, to further identify whether the homologues of GOX 0854 and GOX 0855 catalyze the oxidation of 2,3-butanediol in *G. oxydans* DSM 2003, deletion and function analysis of the corresponding genes should be conducted in successive studies.

### Optimal pH for acetoin production

To increase the efficiency of acetoin production, the bioconversion conditions of *G. oxydans* DSM 2003 were optimized. The effects of the pH (5.5 to 7.5) of the culture medium on growth of *G. oxydans* DSM 2003, 2,3-butanediol utilization, and acetoin production were investigated in 300 mL shake flasks containing 50 mL medium with approximately 10 g/L 2,3-butanediol.

**Figure 3:**
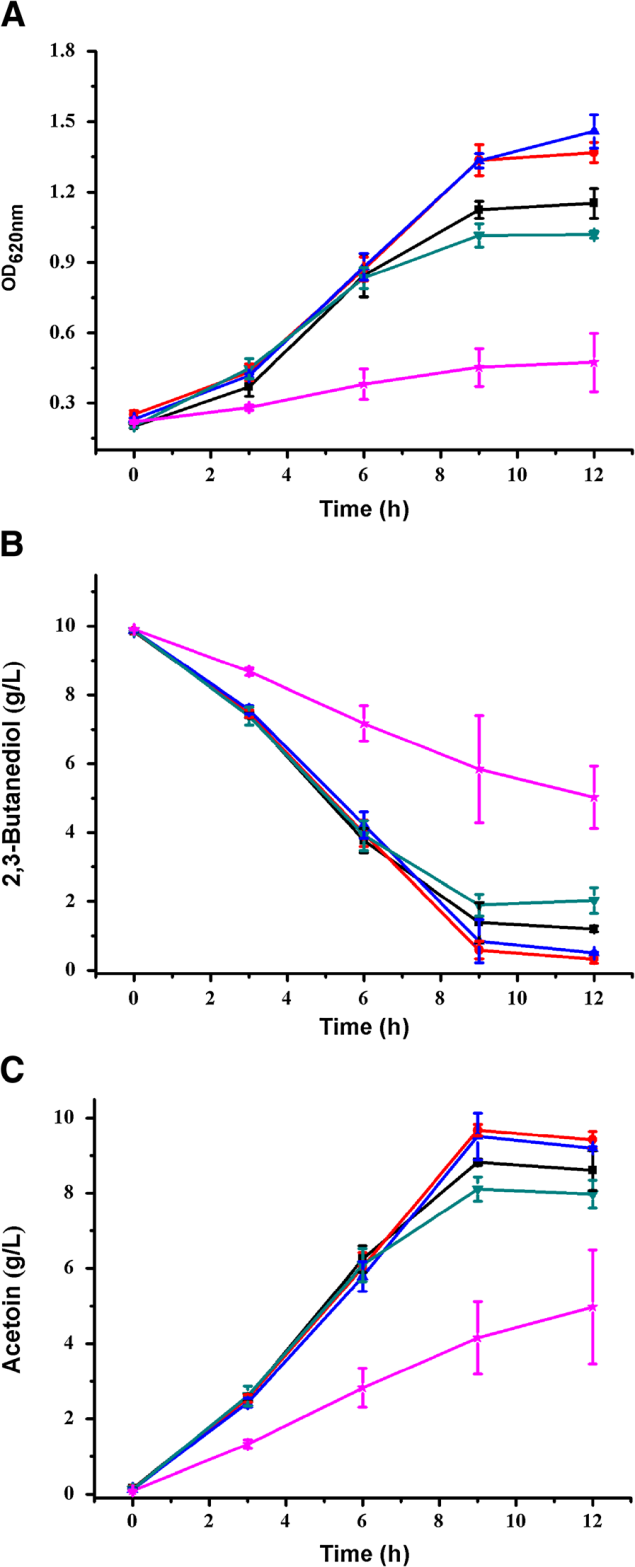
**Effect of pH on acetoin production by *G. oxydans *DSM 2003. (A)** Biomass; **(B)** 2,3-butanediol; and **(C)** acetoin. The experiments were conducted in 300 mL shake flasks containing 50 mL of medium at 30°C. The pH was adjusted at 5.5 (black square), 6.0 (red circle), 6.5 (blue triangle), 7.0 (dark green down-pointing triangle), and 7.5 (pink star).

As shown in Figure 3C, the highest concentration of acetoin was 9.4 g/L when the initial pH of the culture medium was set at 6.0. 2,3-Butanediol (9.8 g/L) was nearly completely depleted during 12 hours of bioconversion (Figure 3B). The product yield was at 98.1% of the theoretical value (1 mol/mol). Consequently, the initial pH of 6.0 was chosen for subsequent bioconversions.

### Optimal temperature for acetoin production

Efficiency of the bioconversion processes is temperature-dependent owing to the strict dependence of enzymatic activity and cellular maintenance upon temperature. In this study, the effects of temperature (16°C, 25°C, 30°C, and 35°C) on cell growth, acetoin production, and 2,3-butanediol utilization were also examined.

**Figure 4:**
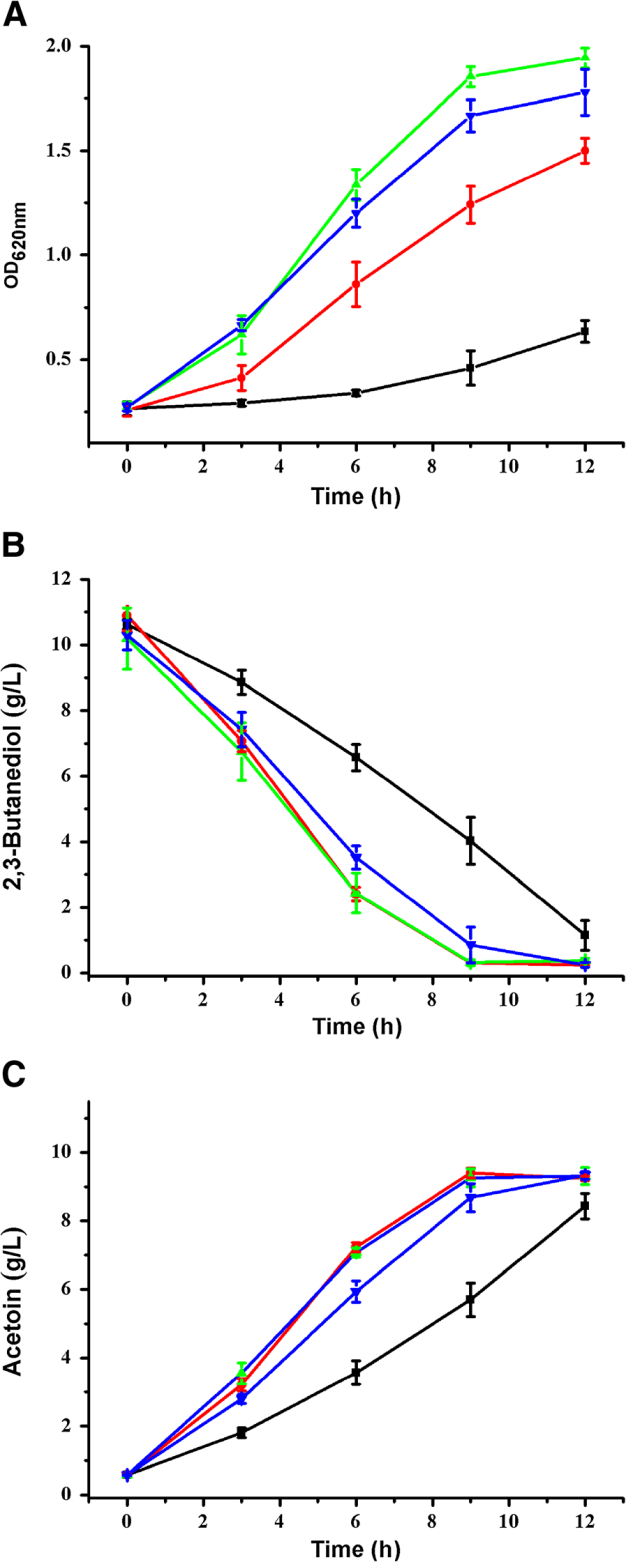
**Effect of temperature on acetoin production by *G. oxydans *DSM 2003. (A)** Biomass; **(B)** 2,3-butanediol; and **(C)** acetoin. The experiments were conducted in 300 mL shake flasks containing 50 mL of medium at pH 6.0. The temperature was adjusted at 16°C (black square), 25°C (red circle), 30°C (green triangle), and 35°C (blue down-pointing triangle).

As shown in Figure 4A and Figure 4C, the best growth of *G. oxydans* DSM 2003 and yield of acetoin were obtained when the temperature was maintained at 30°C. Since the *G. oxydans* strain could not grow at a temperature higher than 37°C, a temperature of 30°C was chosen for subsequent bioconversions.

### Optimal 2,3-butanediol concentration for acetoin production

To study the effect of the initial 2,3-butanediol concentration on acetoin production, various concentrations of 2,3-butanediol were utilized by *G. oxydans* DSM 2003 in batch process to produce acetoin. The effects of 2,3-butanediol concentration on cell and acetoin production were examined after 24 hours of bioconversion in 300 mL shake flasks containing 10 g/L, 20 g/L, 40 g/L, 60 g/L, and 80 g/L 2,3-butanediol, respectively.

**Figure 5:**
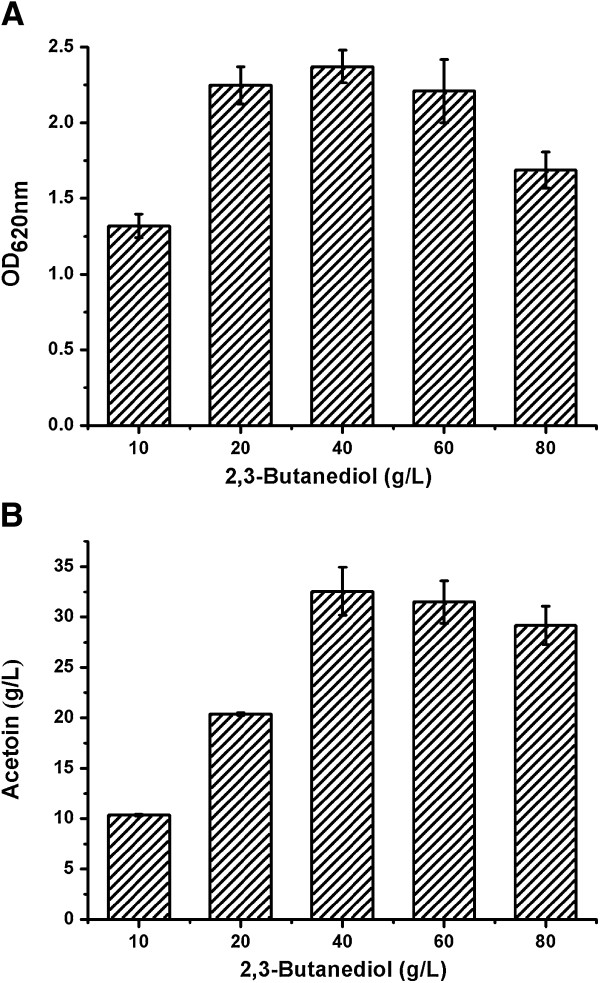
**Effect of 2,3-butanediol concentration on acetoin production by *G. oxydans *DSM 2003. (A)** Biomass; and **(B)** acetoin. The experiments were conducted in 300 mL shake flasks containing 50 mL of medium at pH 6.0 and 30°C. The concentrations of 2,3-butanediol concentration were adjusted at 10 g/L, 20 g/L, 40 g/L, 60 g/L, and 80 g/L.

As shown in Figure 5A, cell density increased with the 2,3-butanediol concentrations to 40 g/L, and then decreased. The production of acetoin increased significantly with an increase of 2,3-butanediol concentrations from 10 g/L to 40 g/L (Figure 5B). When the 2,3-butanediol concentration was over 40 g/L, both cell density and acetoin concentration decreased sharply. This result showed that the high initial substrate concentration would affect the metabolism of strain *G. oxydans* DSM 2003. Thus, 2,3-butanediol at a concentration of 40 g/L was chosen for subsequent studies.

### Batch bioconversion under optimum conditions

Combining the results mentioned above, an optimal system for the production of acetoin from 2,3-butanediol was developed. Bioconversion was conducted at 30°C in 300 mL shake flasks containing 50 mL medium. The medium consisted of 20 g/L yeast extract, 1.5 g/L (NH_4_)_2_SO_4_, 1.5 g/L KH_2_PO_4_, 0.5 g MgSO_4_ 7H_2_O, and 40 g/L 2,3-butanediol. The pH was maintained at 6.0.

**Figure 6:**
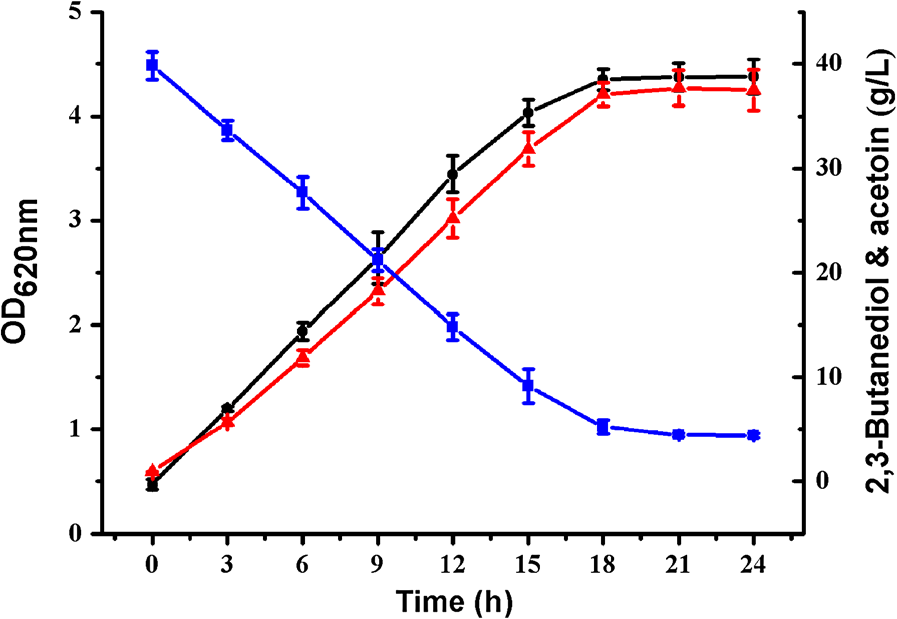
**Time course of batch bioconversion of acetoin from 2,3-butanediol.** The bioconversion was carried out at 30°C in 300 mL shake flasks containing 50 mL of medium at pH 6.0. The initial 2,3-butanediol concentration used was 40 g/L. Biomass (black circle); 2,3-butanediol (blue square); and acetoin (red triangle).

**Figure 7:**
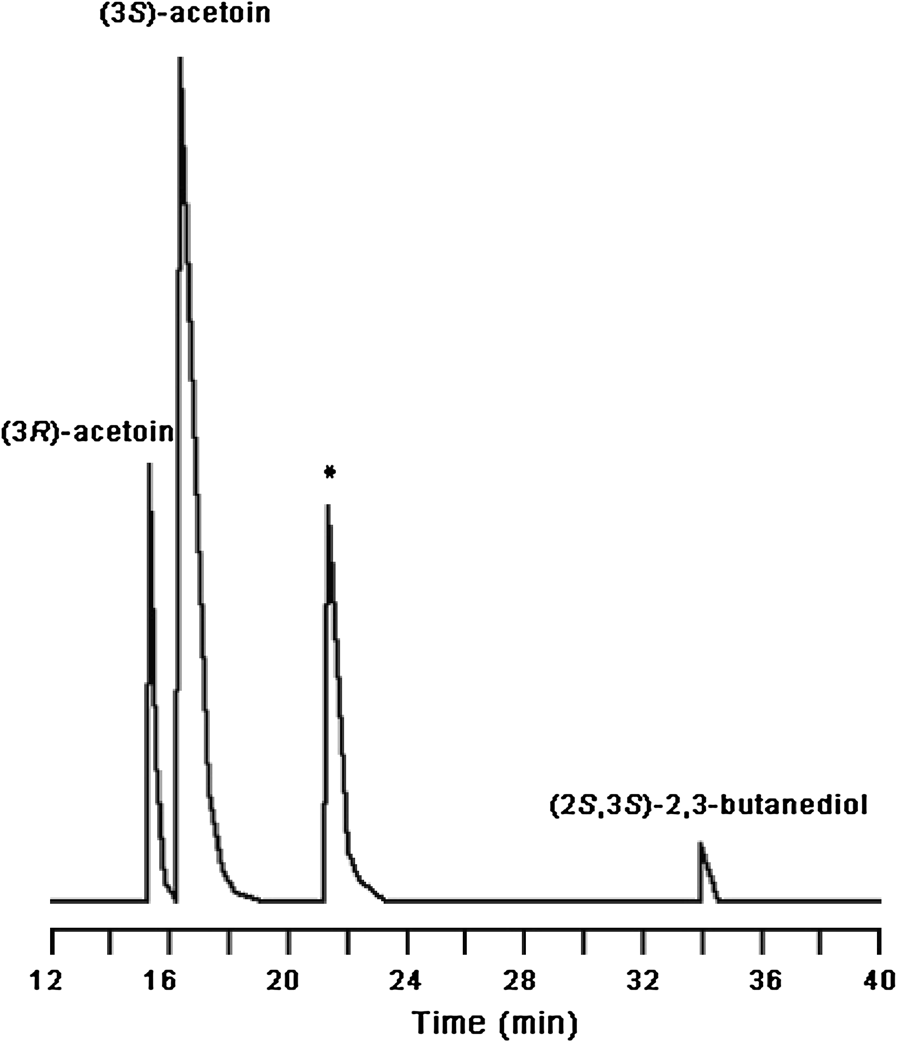
**Gas chromatography (GC) analysis of products of the bioconversion process.** *Isoamyl alcohol was used as the internal standard.

As shown in Figure 6, 37.5 g/L acetoin was obtained from 40 g/L 2,3-butanediol after 24 hours of bioconversion. No other products were detected during the bioconversion process. The yield of acetoin was at 95.9% of the theoretical value. The ratio of (3*R*)-acetoin and (3*S*)-acetoin produced by strain *G. oxydans* DSM 2003 was analyzed by GC, which were 20.0% of (3*R*)-acetoin and 80.0% of (3*S*)-acetoin, respectively (Figure 7). Since *G. oxydans* DSM 2003 catalyzes 2,3-butanediol oxidation with stereoselectivity, the ratio of (3*R*)-acetoin and (3*S*)-acetoin would be controlled by the stereoisomer of 2,3-butanediol used.

### Fed-batch bioconversion

Efficient fed-batch bioconversion could enhance the concentrations of the target products. To achieve a higher product concentration, a fed-batch bioconversion was carried out with the optimized bioconversion conditions. The initial 2,3-butanediol concentration was 40 g/L, and 20 g/L of 2,3-butanediol was added at 12, 24, and 36 hours, respectively.

**Figure 8:**
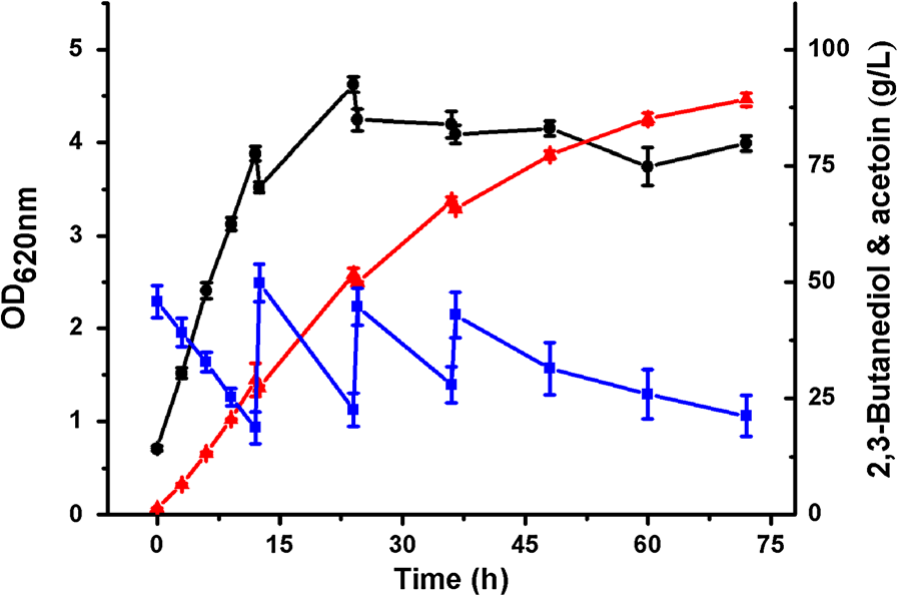
**Time course of fed-batch bioconversion of acetoin from 2,3-butanediol.** The bioconversion was carried out at 30°C in 300 mL shake flasks containing 50 mL of medium at pH 6.0. The initial 2,3-butanediol concentration used was 40 g/L, and 20 g/L of 2,3-butanediol was added at 12, 24, and 36 hours, respectively. Biomass (black circle); 2,3-butanediol (blue square); and acetoin (red triangle).

As shown in Figure 8, a high concentration of 89.2 g/L acetoin was produced from 2,3-butanediol within 72 hours. The acetoin productivity was 1.24 g/L · h with a yield of 0.91 mol/mol 2,3-butanediol. As shown in Table 1, 89.2 g/L of acetoin obtained in this study is the highest acetoin concentration obtained to date.

**Table 1:** Comparison of the acetoin production using different biocatalysts and fermentative strains

	**Substrate**	**Concentration (g/L)**	**Productivity (g/L · h)**	**Yield (mol/mol)**	**Reference**
**Biocatalyst**					
*Escherichia coli* expressing glycerol dehydrogenase	Mixture of *meso*-2,3-butanediol and (2*S*,3*S*)-2,3-butanediol	8.4	0.35	0.86	[20]
*Escherichia coli* strain coexpressed (2*R*,3*R*)-2,3-butanediol dehydrogenase and NADH oxidase	*Meso*-2,3-butanediol	36.7	3.06	0.85	[21]
*Escherichia coli* expressed diacetyl reductase	Diacetyl and glucose	13.5	2.25	0.91	[40]
Purified NADPH-dependent carbonyl reductase and glucose dehydrogenase	Diacetyl and glucose	12.2	9.76	0.85	[41]
**Fermentative strain**					
*Serratia marcescens* H32 expressed NADH oxidase	Sucrose	75.2	1.88	0.78	[16]
*Klebsiella pneumoniae* expressed NADH oxidase	Glucose	25.9	0.32	0.16	[18]
*Geobacillus* strain XT15	Glucose	7.7	0.16	0.24	[17]
*Bacillus licheniformis* MEL09	Glucose	41.3	1.15	0.84	[42]
*Bacillus subtilis* JNA310	Glucose	42.2	0.32	0.57	[43]
*Bacillus subtilis* moderately expressed the transcriptional regulator AlsR	Glucose	41.5	0.43	0.71	[44]
*Bacillus subtilis* JNA-UD-6	Glucose	53.9	0.37	0.74	[45]
*Klebsiella oxytoca* M1	Glucose	27.4	0.57	0.78	[46]
*Bacillus subtilis* CICC10025	Glucose	35.4	0.63	0.83	[47]
*Gluconobacter oxydans* DSM 2003	Mixture of *meso*-2,3-butanediol, (2*R*,3*R*)-2,3-butanediol, and (2*S*,3*S*)-2,3-butanediol	89.2	1.24	0.91	This study

Several biotechnological routes including enzymatic or whole-cell conversion methods [20,21,40,41] and fermentative technologies [18,19,42-47] have been used to produce acetoin (Table 1). Among all of the reported biotechnological processes, Sun *et al*. obtained the highest acetoin concentration of 75.2 g/L with *S. marcescens* H32 with the expression of a water-forming NADH oxidase [16]. However, there were still considerable amounts of 2,3-butanediol generated during the acetoin fermentation process. Efforts have been tried in order to increase acetoin production through further biotransformation of the 2,3-butanediol [43]. Using 2,3-butanediol as the substrate, the recombinant *E. coli* strain that coexpressed (2*R*,3*R*)-2,3-butanediol dehydrogenase and NADH oxidase produced acetoin at a high concentration of 36.7 g/L [21]. On the other hand, diacetyl could also be used as the substrate for acetoin production [40,41]. Acetoin at a concentration of 13.5 g/L was produced from diacetyl by using an *E. coli* strain that expressed stereoselective diacetyl reductase [41].

In this study, we found that *G. oxydans* DSM 2003 is able to produce considerable quantities of acetoin using 2,3-butanediol as the carbon source. Both concentration and yield of acetoin produced by the novel process are new records for acetoin production. Although 2,3-butanediol could be easily produced by fermentation, its large-scale microbial production requires development of efficient derivative processes. Thus, the method presented in this study would not only provide a promising process for acetoin production, but would also expand the utilization of 2,3-butanediol produced from biomass.

## Conclusions

An efficient process for acetoin production from 2,3-butanediol was developed by using *G. oxydans* DSM 2003. All three stereoisomers of 2,3-butanediol could be oxidized into acetoin by the strain. Under optimal conditions, the bioconversion process exhibited rather high concentration (89.2 g/L), productivity (1.24 g/L · h), and yield (91.2%) of acetoin. The results of this study suggest that production of acetoin using 2,3-butanediol can serve as a choice for the derivative of industrially produced 2,3-butanediol.

## Materials and methods

### Materials

(2*R*,3*R*)-2,3-Butanediol (98.0%), (2*S*,3*S*)-2,3-butanediol (99.0%), and *meso*-2,3-butanediol (98.0%) were purchased from Acros (Geel, Belgium). The mixture of 2,3-butanediol (76.1% *meso*-2,3-butanediol, 15.9% (2*R*,3*R*)-2,3-butanediol, and 8.0% (2*S*,3*S*)-2,3-butanediol) was obtained from Sinopharm (Beijing, China). Racemic acetoin, diacetyl, phenazine methosulfate (PMS), and DCPIP were purchased from Sigma. All other chemicals were of analytical grade and commercially available.

### Microorganism and culture conditions

*G. oxydans* DSM 2003 (Deutsche Sammlung von Mikroorganismen und Zellkulturen (DSMZ), Braunschweig, Germany) was used in this study. The strain was cultured in a medium containing 20 g yeast extract, 1.5 g (NH_4_)_2_SO_4_, 1.5 g KH_2_PO_4_, and 0.5 g MgSO_4_ 7H_2_O in 1 L of distilled water. This medium was supplemented with 2,3-butanediol, glucose, glycerol, or sorbitol as the carbon source. The flask experiment was conducted in 300 mL shake flasks containing 50 mL fresh medium.

### Whole-cell DCPIP assay of the membrane-bound 2,3-butanediol dehydrogenase

For the assay of the membrane-bound 2,3-butanediol dehydrogenase, whole cells of *G. oxydans* DSM 2003 were concentrated to OD_620nm_ 4.0 via centrifugation at 4,000 × g for 5 minutes. The concentrated cells were washed in 10 mL 67 mM phosphate buffer (pH 7.4), resuspended in the same buffer and then immediately used. Activity of 2,3-butanediol dehydrogenase was determined at 30°C in 1 mL of 67 mM phosphate buffer, pH 7.4, 0.2 mM PMS, 0.2 mM DCPIP, and whole cells of *G. oxydans* DSM 2003 (final OD_620nm_ of 0.2). The reaction was started by addition of 25 mM *meso*-2,3-butanediol, (2*R*,3*R*)-2,3-butanediol, or (2*S*,3*S*)-2,3-butanediol [45]. The rate of DCPIP reduction was determined by measuring the absorbance changes at 600 nm [48]. An extinction coefficient of 21,300 for DCPIP was used for the rate calculation. One unit of oxidation activity was defined as 1 μmol substrate oxidized per minute as determined by reduction of 1 μmol DCPIP.

### Optimization of bioconversion conditions

For the optimization of bioconversion conditions, the culture medium of 50 mL in 300 mL shake flasks were used with variation as follows: the pH values were 5.5 to 7.5, temperatures were 16°C to 35°C, and 2,3-butanediol concentrations were 10 g/L to 80 g/L. Bioconversion was carried out for 12 hours and then the reaction mixture was centrifuged. The resultant supernatant was analyzed for 2,3-butanediol and acetoin by GC.

### Analytical methods

Samples were withdrawn periodically and centrifuged at 12,000 × g for 10 minutes. The growth of *G. oxydans* DSM 2003 was determined by monitoring the absorbance at 620 nm using a spectrophotometer (Lengguang 721, Shanghai Precision & Scientific Instrument Co Ltd, Shanghai, China) after an appropriate dilution. The concentrations of 2,3-butanediol and acetoin were analyzed by GC (Varian 3800, Varian, Walnut Creek, CA, USA) with the method described by Xiao *et al*. [21]. The GC system was equipped with a 30 m SPB-5 capillary column (0.32 mm inside diameter, 0.25 μm film thickness; Supelco, Bellefonte, PA, USA) and a flame ionization detector. The injector and detector temperatures were both 280°C. The column oven temperature was maintained at 40°C for 3 minutes, and then raised to 240°C at a rate of 20°C/minute. The injection volume was 1 μL. The calibration curve was used to calculate the concentration of the products. The concentration of diacetyl was measured by HPLC (Agilent 1100 series, Hewlett-Packard, Waldbronn, Germany) equipped with an Aminex HPX-87H column (300 × 7.8 mm) (Bio-Rad, Hercules, CA, USA) and a refractive index detector [49]. The analysis was performed with a mobile phase of 10 mM H_2_SO_4_ at a flow rate of 0.4 mL/minute and at 55°C.

## Abbreviations

DCPIP: 2,6-dichlorophenolindophenol; DSMZ: Deutsche Sammlung von Mikroorganismen und Zellkulturen; GC: Gas chromatography; HPLC: High-performance liquid chromatography; PMS: Phenazine methosulfate.

## Competing interests

The authors declare that they have no competing interests.

## Authors’ contributions

CG and CM participated in the design of the study. XW and ML executed the experimental study. KL and LZ analyzed the data. CG, CM, and PX contributed reagents and materials. CG, CM, and PX wrote and revised the manuscript. All authors read and approved the final manuscript.

## Supplementary Material

Additional file 1: Figure S1 Time course of *G. oxydans* DSM 2003 growth in the medium containing **(A)** 20 g/L yeast extract or **(B)** 10 g/L glycerol. Click here for file

Additional file 2: Figure S2 Analysis of the utilization of acetoin by *G. oxydans* DSM 2003. Click here for file

Additional file 3: Table S1 Effects of carbon sources on the activities of 2,3-butanediol dehydrogenases. Click here for file
