# Supplementary material for: Efficient bioconversion of 2,3-butanediol into acetoin using Gluconobacter oxydans DSM 2003
Source: Biotechnol Biofuels. 2013 Oct 31;6:155. doi: 10.1186/1754-6834-6-155 (PMC4177140; doi:10.1186/1754-6834-6-155)
Supplement: Additional file 1: Figure S1 — Time course of G. oxydans DSM 2003 growth in the medium containing (A) 20 g/L yeast extract or (B) 10 g/L glycerol. [file 1754-6834-6-155-S1.doc]

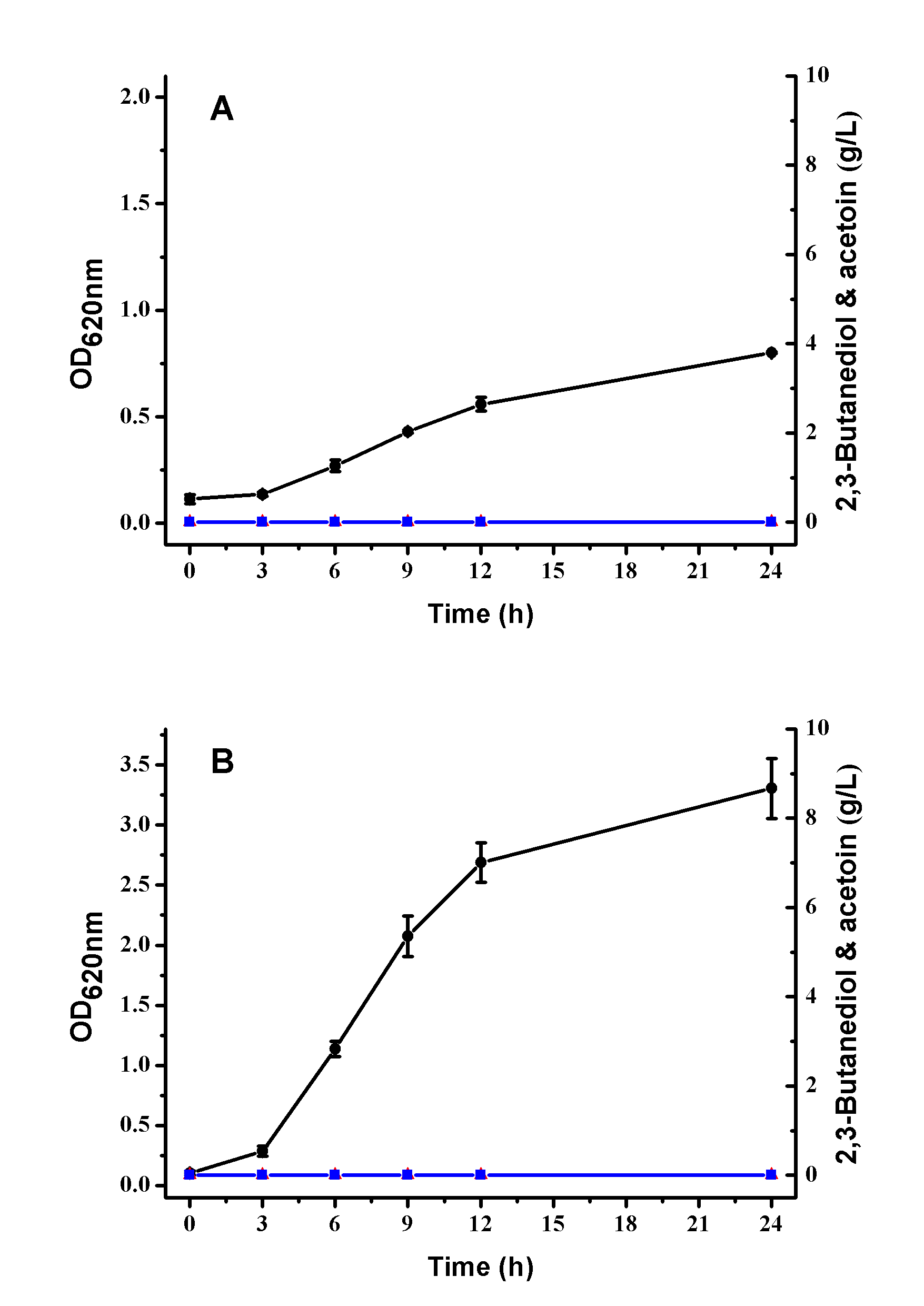


**Additional file 1 – Figure S1 Time course of *G. oxydans* DSM2003 growth in the medium containing 20 g/L yeast extract (A) or 10 g/L glycerol (B).**

The experiments were conducted in 300-mL shake flasks containing 50 mL of medium at 30^o^C. Biomass (●), 2,3-Butanediol (■), Acetoin (▲).
