# Supplementary material for: Efficient bioconversion of 2,3-butanediol into acetoin using Gluconobacter oxydans DSM 2003
Source: Biotechnol Biofuels. 2013 Oct 31;6:155. doi: 10.1186/1754-6834-6-155 (PMC4177140; doi:10.1186/1754-6834-6-155)
Supplement: Additional file 2: Figure S2 — Analysis of the utilization of acetoin by G. oxydans DSM 2003. [file 1754-6834-6-155-S2.doc]

**Additional file 2 – Figure S2 Analysis of the utilization of acetoin by *G. oxydans* DSM2003.**

A, HPLC analysis of authentic acetoin and diacetyl. B, HPLC analysis of the reaction mixture with acetoin as substrate after biotransformation.
