# Supplementary material for: Efficient bioconversion of 2,3-butanediol into acetoin using Gluconobacter oxydans DSM 2003
Source: Biotechnol Biofuels. 2013 Oct 31;6:155. doi: 10.1186/1754-6834-6-155 (PMC4177140; doi:10.1186/1754-6834-6-155)
Supplement: Additional file 3: Table S1 — Effects of carbon sources on the activities of 2,3-butanediol dehydrogenases. [file 1754-6834-6-155-S3.doc]

## Table S1 Effects of carbon sources on the activities of the membrane-bound 2,3-butanediol dehydrogenases

| Carbon source | Specific activities 2,3-butanediol dehydrogenases (mU/g) ^a^ | | |
| --- | --- | --- | --- |
|  | *meso*-2,3-butanediol | (2*R*,3*R*)-2,3-butanediol | (2*S*,3*S*)-2,3-butanediol |
| 2,3-Butanediol | 73.2 ± 9.3 | 70.2 ± 6.0 | 83.2 ± 6.0 |
| Glucose | 60.1 ± 6.7 | 60.0 ± 4.9 | 75.3 ± 2.1 |
| Glycerol | 59.1 ± 9.2 | 60.6 ± 5.5 | 79.0 ± 5.0 |
| Sorbitol | 71.9 ± 6.7 | 71.0 ± 8.9 | 115.8 ± 2.0 |

^a^ Values are the average ± SD of three separate determinations.

^b^ The specific activities of 2,3-butanediol dehydrogenase were defined on the basis of gram dry cell weight of whole cells.
